# Supplementary material for: Gold bipyramids as a promising alternative to gold nanorods for analytical and biomedical applications
Source: Beilstein J Nanotechnol. 2026 Aug 25;17:1170–84. doi: 10.3762/bjnano.17.80 (PMC13550847; doi:10.3762/bjnano.17.80)
Supplement: Supplementary file 1 [file Beilstein_J_Nanotechnol-17-1170-s001.pdf]

BEILSTEIN JOURNAL OF NANOTECHNOLOGY

Supporting Information

for

Gold bipyramids as a promising alternative to gold nanorods for analytical and biomedical applications

Andrey M. Burov, Sergey V. Zarkov, Arina V. Drozd, Igor V. Borisov, Elena G. Zavyalova and Nikolai G. Khlebtsov

Beilstein J. Nanotechnol. **2026,** *17,* 1170–1184. doi:10.3762/bjnano.17.80

Additional experimental and calculation data

License and Terms: This is a supporting information file under the terms of the Creative Commons Attribution License (https://creativecommons.org/licenses/by/4.0). Please note that the reuse, redistribution and reproduction in particular requires that the author(s) and source are credited and that individual graphics may be subject to special legal provisions.
The license is subject to the *Beilstein Journal of Nanotechnology* terms and conditions: (https://www.beilstein-journals.org/bjnano/terms)

Section S1. Additional TEM images and particle statistics

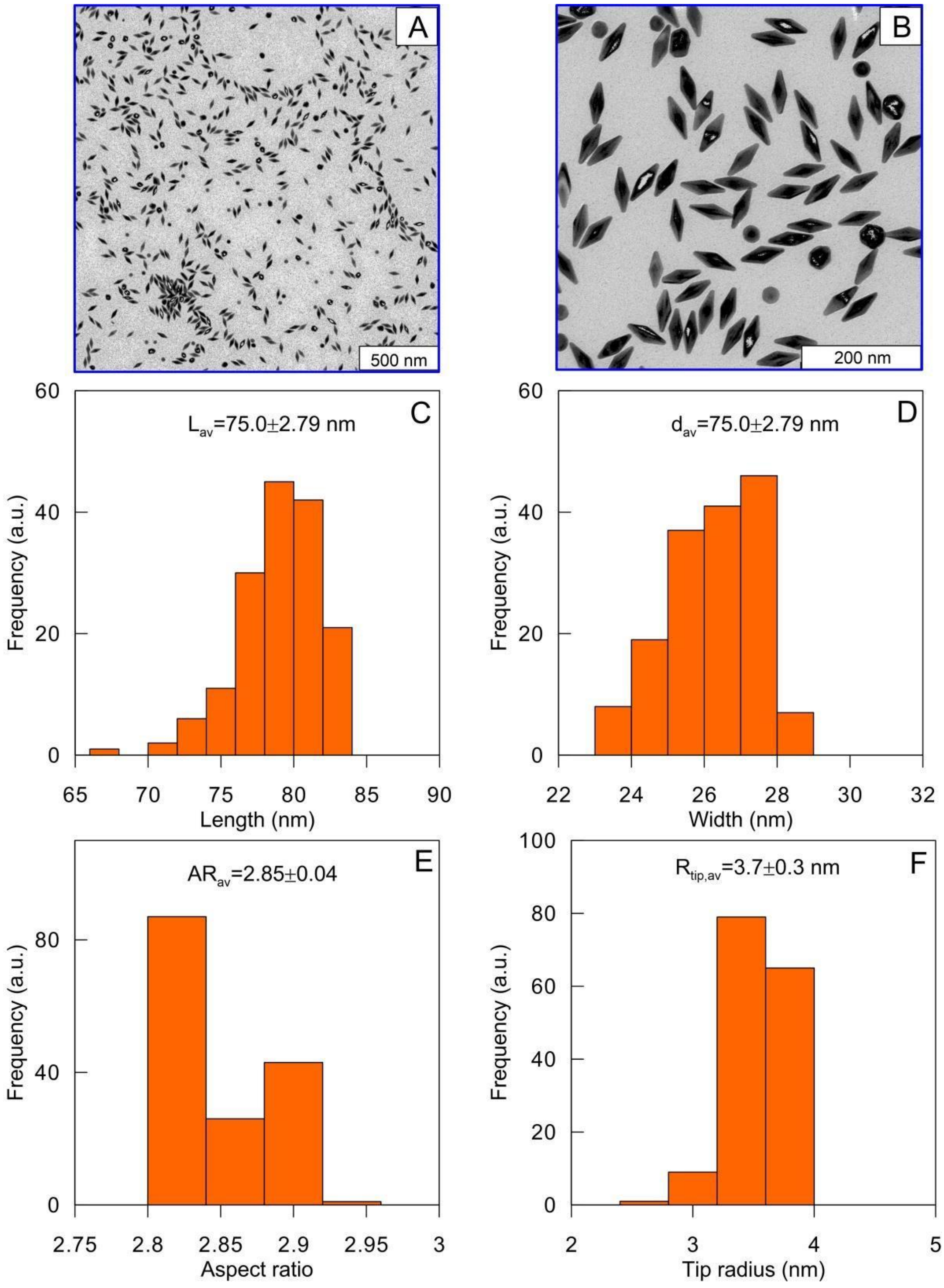

Figure S1: Additional TEM images of the BP-735 sample (A, B) and corresponding histograms showing the distributions of length (C), width (D), aspect ratio (E), and tip radius (F). Scale bars: 500 nm (A) and 200 nm (B). Mean values and standard deviations are indicated in each panel.

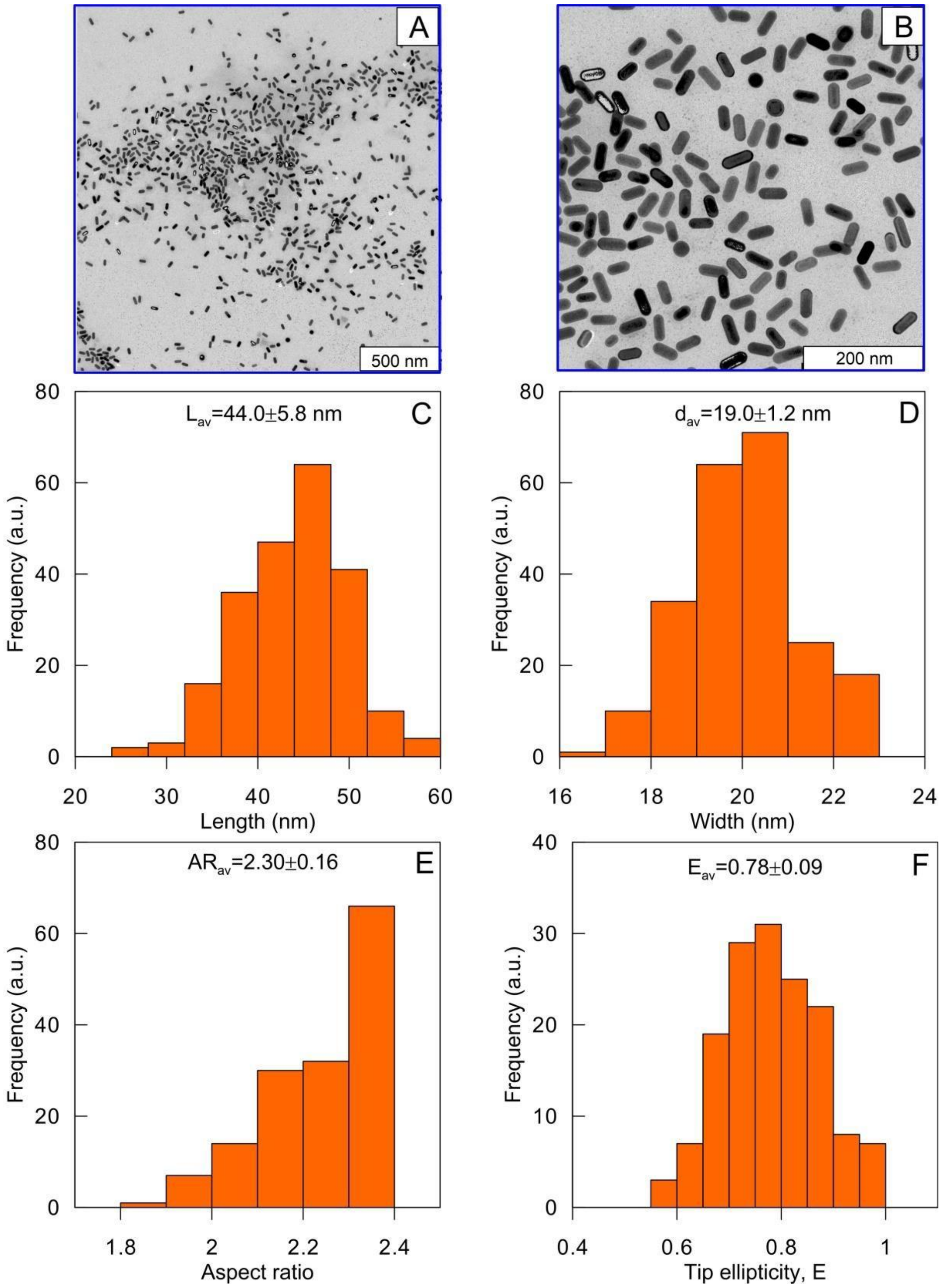

Figure S2: Additional TEM images of the NR-640 sample (A, B) and corresponding histograms of length (C), width (D), aspect ratio (E), and tip ellipticity (F). Scale bars: 500 nm (A) and 200 nm (B). Mean values and standard deviations are shown in each panel.

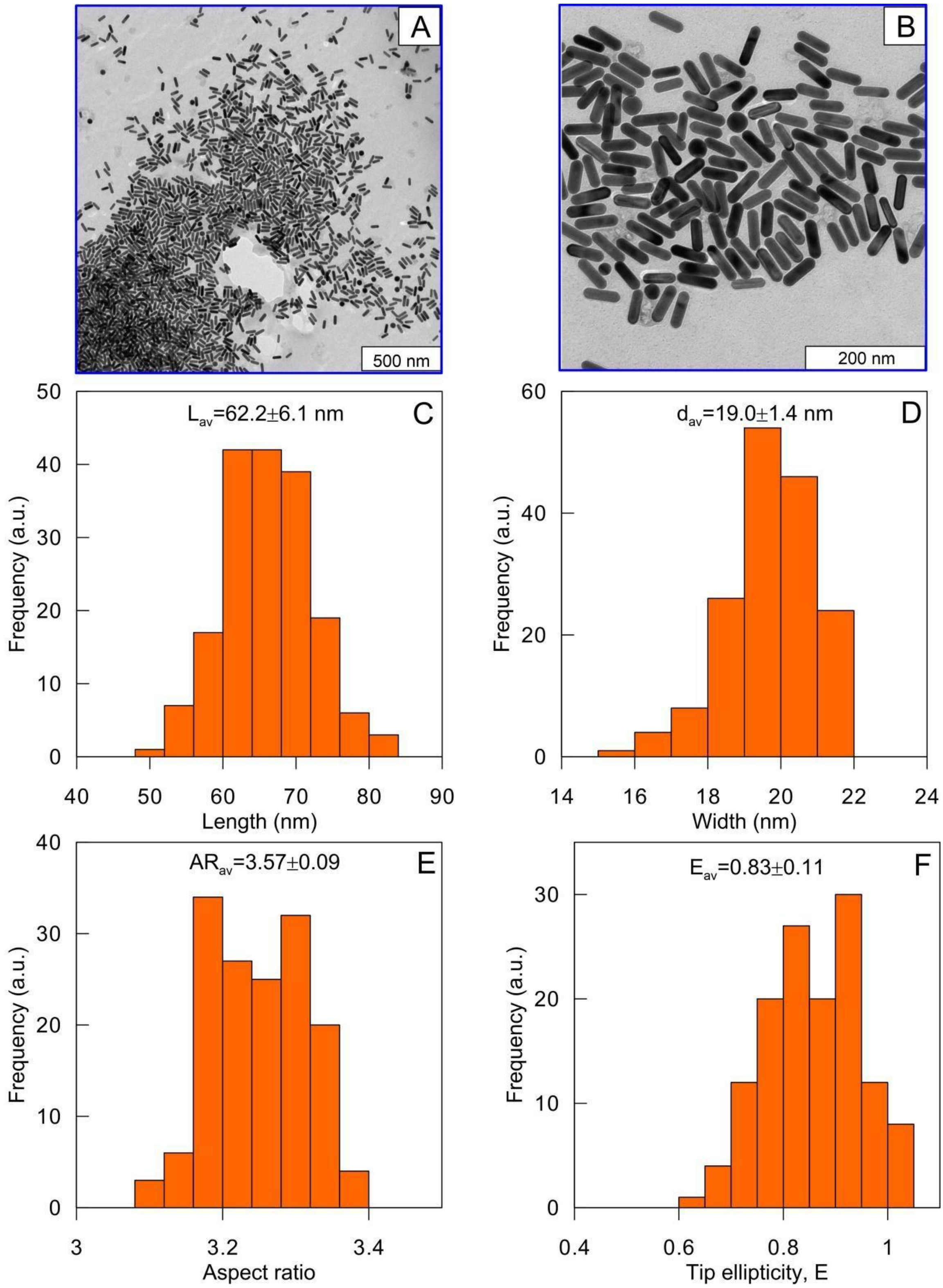

Figure S3: Additional TEM images of the NR-735 sample (A, B) and corresponding histograms of length (C), width (D), aspect ratio (E), and tip ellipticity (F). Scale bars: 500 nm (A) and 200 nm (B). Mean values and standard deviations are shown in each panel.

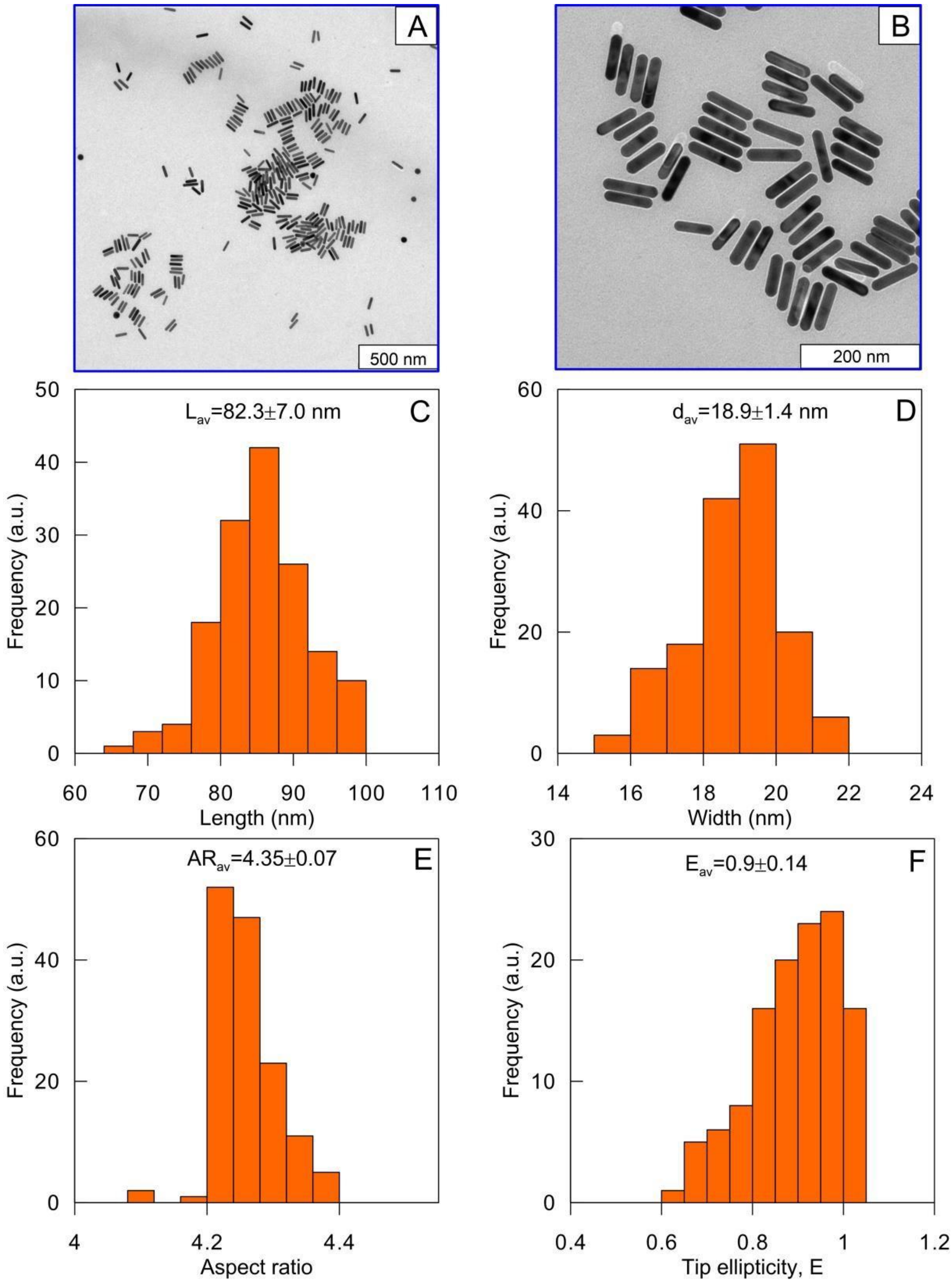

Figure S4: Additional TEM images of the NR-844 sample (A, B) and corresponding histograms of length (C), width (D), aspect ratio (E), and tip ellipticity (F). Scale bars: 500 nm (A) and 200 nm (B). Mean values and standard deviations are shown in each panel.

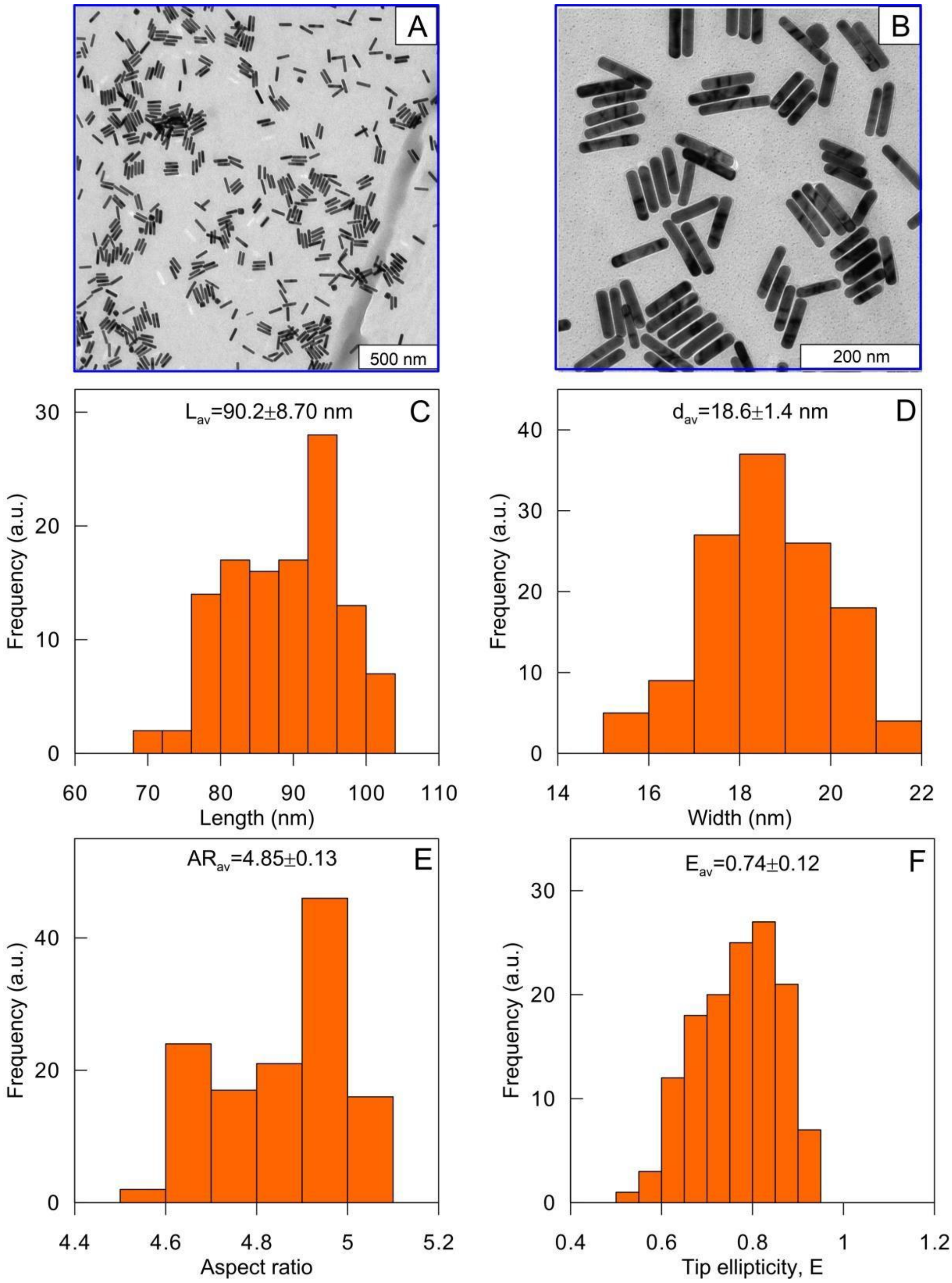

Figure S5: Additional TEM images of the NR-945 sample (A, B) and corresponding histograms of length (C), width (D), aspect ratio (E), and tip ellipticity (F). Scale bars: 500 nm (A) and 200 nm (B). Mean values and standard deviations are shown in each panel.

Section S2. Comparison of extinction spectra calculated by COMSOL and the T-matrix method

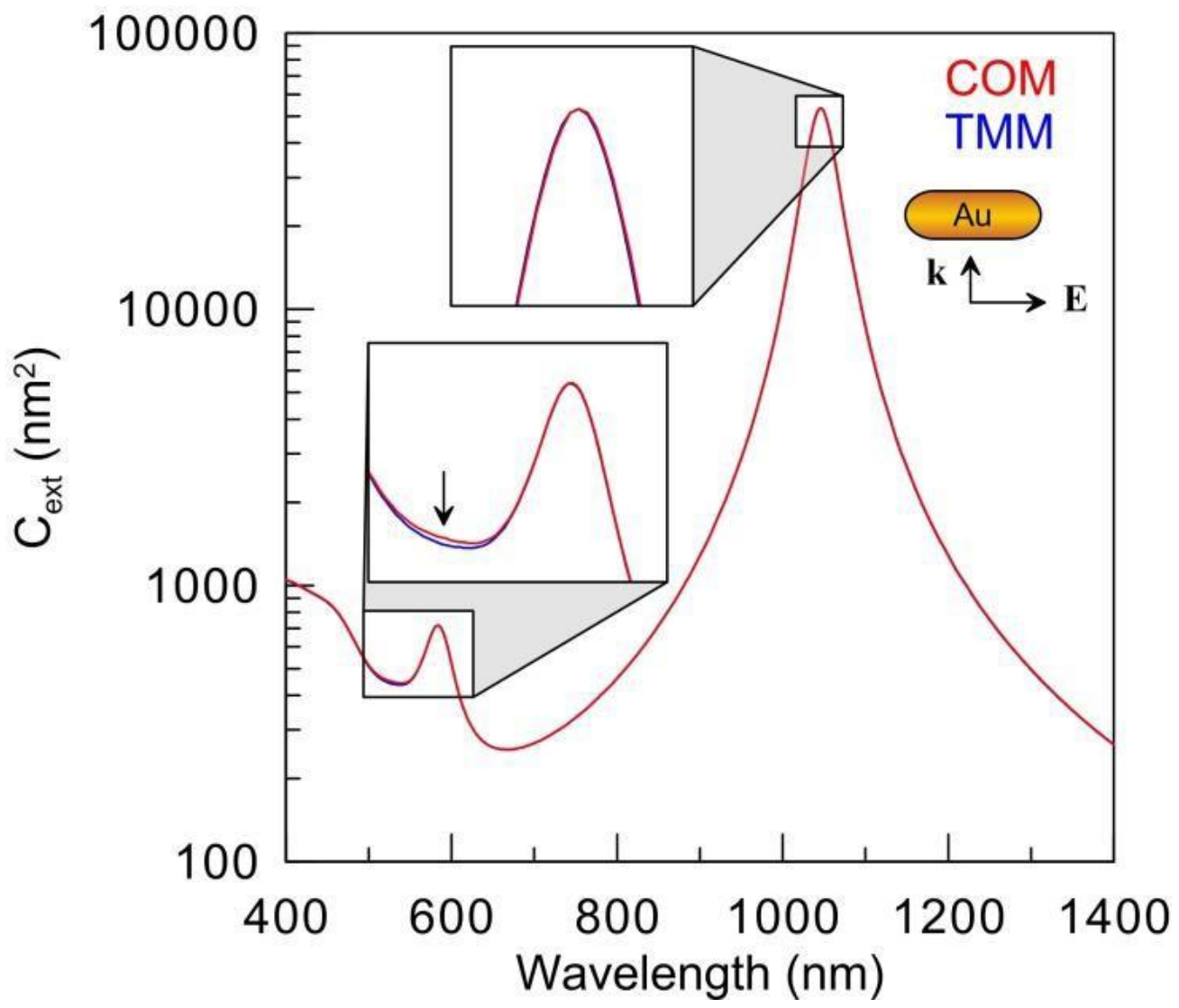

Figure S6: Comparison of extinction spectra calculated using COMSOL (COM, red curve) and the T-matrix method (TMM, blue curve) for longitudinal excitation of an AuNR with a length of 90 nm and a diameter of 15 nm. The two spectra are in excellent agreement, except for a slight discrepancy near 550 nm. A small peak near 580 nm corresponds to the quadrupole excitation.

Section S3. Calculations of the average near field parameters

According to Eq. (11) of the main text, one has to calculate integrals over the particle outer surface

$$\langle |E(\lambda_{PR})| \rangle = \frac{1}{S} \int_S |E(\lambda_{PR})/E_0| \, \mathrm{d}S, \tag{S1}$$

Due to axial symmetry, this integral can be reduced to the 1D integration over the main particle cross-section across the symmetry axis. Figure S7 gives a typical representation of the local field distribution across such a section for a nanorods and bipyramid example. The particle length, width, and aspect ratios, correspond to those indicated in Table 1. For bipyramids, the tip and base rounding radii are 4 nm and 12 nm, respectively.

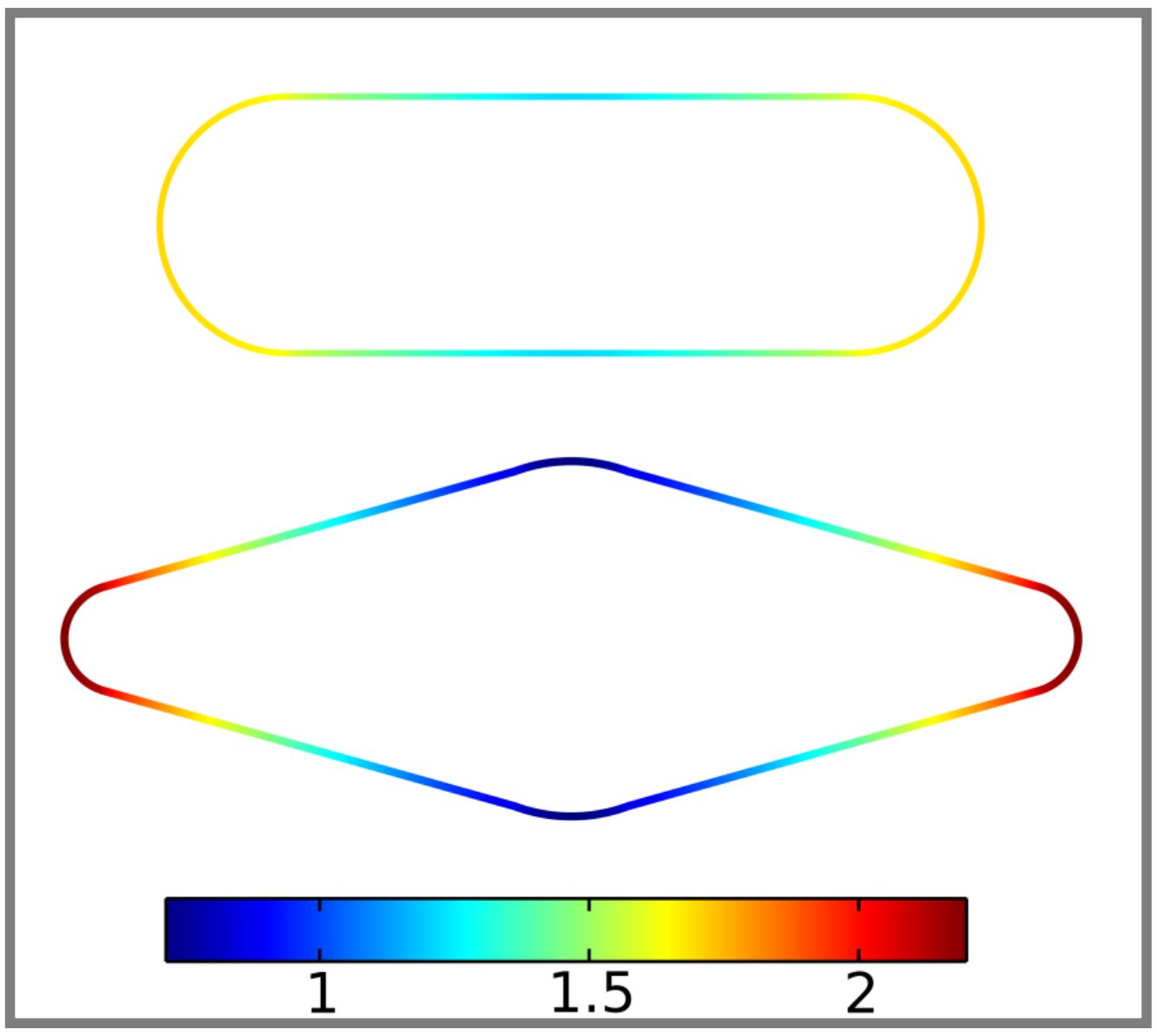

Figure S7: Distribution of $\log_{10} |\boldsymbol{E}/\boldsymbol{E}_0|$ along the boundary of the nanorod (top) and the bicone (bottom) in a medium (nm = 1.334) at the longitudinal resonance excitation. The inscribed bicone represent the corresponding bipyramid shape [1]. The particle parameters and plasmon resonance wavelengths are indicated in Table 1 of the main text for the NR-735 and BC-753 samples.

References

1. Montaño-Priede, J. L.; Sánchez-Iglesias, A.; Mezzasalma, S. A.; Sancho-Parramon, J.; Grzelczak, M. *J. Phys. Chem. Lett*. **2024**, *15*, 3914–3922
